# Quantification of a peptide standard using the intrinsic fluorescence of tyrosine

**DOI:** 10.1007/s00216-016-9334-1

**Published:** 2016-02-15

**Authors:** George W. Preston, David H. Phillips

**Affiliations:** Analytical & Environmental Sciences Division, MRC-PHE Centre for Environment & Health, King’s College London, Franklin-Wilkins Building, 150 Stamford Street, London, SE1 9NH UK

**Keywords:** Amino acids/peptides, Genomics/proteomics, HPLC, Fluorescence/luminescence

## Abstract

Absolute quantification of peptides is typically achieved using amino acid analysis, elemental analysis or derivatisation chemistry. Impurities, if present, may be accounted for using analytical high-performance liquid chromatography (HPLC) with detection of the peptide bond ultraviolet (UV) absorbance. To do this, peak areas from a UV chromatogram are used to estimate percentage purity on a mass basis, and this purity value is used as a correction. However, because the approach assumes that UV absorbance is uniformly proportional to mass, the result may be only semi-quantitative. Here, an alternative approach involving HPLC with detection of intrinsic tyrosine fluorescence is described. The fluorescence properties of a 21-residue synthetic peptide corresponding to an *S*-carbamidomethylated tryptic fragment of human serum albumin were characterised, and a method involving quantification relative to a non-peptidic calibrant, *N*-acetyl-l-tyrosine ethyl ester, was established. The method was used to quantify the thiol form of the peptide, and the results were compared with a parallel analysis involving derivatisation of the same material with Ellman’s reagent. When differences in fluorescence response (analyte versus calibrant) were accounted for, the measurements obtained via the two methods were in good agreement. Contributions from peptidic impurities were also considered, and their influence on the validity of the conclusions was evaluated. Despite some ambiguities introduced by the impurities, and the identification of some other potential sources of error, the results demonstrate that use of Tyr fluorescence is a promising solution to the challenging problem of absolute peptide quantification.

**Electronic supplementary material:**

The online version of this article (doi:10.1007/s00216-016-9334-1) contains supplementary material, which is available to authorized users.

**Graphical Abstract:**
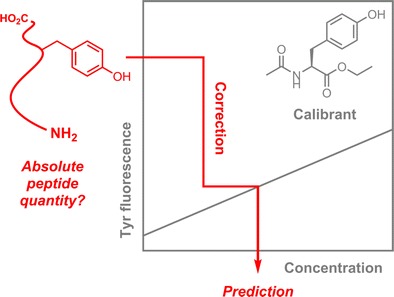
A low-molecular-weight tyrosine derivative was used as a generic fluorescence calibrant for the quantification of a 21-residue peptide

## Introduction

For quantitative studies employing synthetic peptides (e.g. as ligands [1] or standards [2]), an assay is useful for determining the starting peptide concentration. Typical assays involve degradation analysis (e.g. elemental analysis [2, 3] or amino acid analysis [2, 3]) or chemical derivatisation (e.g. reaction with fluorescamine [4]) followed by spectroscopic determination. Alternatively, if present, the intrinsic UV-absorbing properties of tyrosine (Tyr) and/or tryptophan can be exploited to achieve a similar goal [1]. Of course, if sufficient material is available, quantification might be achieved by simple weighing, but the contributions of peptidic [5] and non-peptidic [6] impurities must be taken into account. Purity must also be considered when using degradation analysis or spectroscopy because peptidic impurities can contribute additionally to the observed signal. Analytical high-performance liquid chromatography (HPLC) with short-wavelength UV detection (HPLC-UV) is the preferred method for assessing purity because of its ability to detect and resolve impurities [6]. The resulting chromatogram is integrated, and the peak area due to the peptide of interest is expressed as a percentage of the total area for all peaks. This is undoubtedly a convenient method, but the estimates it generates cannot necessarily be applied directly to the material’s mass; to do so would require the peak area to be uniformly proportional to mass for all components (i.e. peptide of interest, peptidic impurities and non-peptidic impurities), and this assumption is not always valid [7].

It would be useful if a peptide could be quantified directly, without having to correct for impurities that may or may not have been adequately characterised. It would also be useful if the reference standard for the method were non-peptidic, because this would allow the uncertainties described above to be completely eliminated from the analysis. To these ends, some promising results have been obtained using HPLC with online chemical oxidation and mass spectrometry [7] or using HPLC with detection of labelled [8] or unlabelled [9] peptides by inductively coupled plasma mass spectrometry (ICP-MS). These methods are sensitive but might not be appropriate in all cases: the chemical oxidation method requires specialised instrumentation, whilst ICP-MS can only be used for unlabelled peptides if they contain phosphorus, sulfur or selenium.

As an alternative, we explored the possibility of using HPLC with detection of intrinsic Tyr fluorescence (HPLC-FD_Tyr_). By analogy with the ICP-MS approaches, we envisaged benefits from employing a generic (i.e. non-peptidic) fluorescence calibrant for quantifying peptides. A similar idea, albeit based on UV absorption rather than fluorescence, underlies methods for predicting the molar extinction coefficients (*ε*) of proteins [10, 11]: using only sequence information and the *ε* values of model compounds, *ε* values of proteins have been predicted with a good degree of accuracy. One such small molecule used for this purpose was *N*-acetyl-l-tyrosine ethyl ester (Ac-Tyr-OEt) [11, 12], in which the amino and carboxylic acid groups of Tyr are blocked. In the present work, it was hypothesised that Ac-Tyr-OEt would recapitulate not only the UV-absorbing properties of a Tyr residue in a peptide but also the fluorescence. Furthermore, according to the ‘generic calibrant’ idea, we considered that it might be possible to predict peptide concentrations using a known functional relationship between Ac-Tyr-OEt concentration and fluorescence response.

The method described herein was developed to address a specific need, namely the quantification of a peptide derivative with sequence ALVLIAFAQYLQQCPFEDHVK, in which cysteine (Cys) is carbamidomethylated and valine is fully labelled with ^13^C and ^15^N. This structure, which is henceforth referred to as Cam-iT3, is a labelled analogue of product T3 from tryptic digestion of human serum albumin [13]. Cam-iT3 is used as an internal standard in a recently developed adductomics workflow [13], and is prepared by reacting the corresponding peptide thiol (iT3) with iodoacetamide. Results for both Cam-iT3 and unmodified iT3 highlight potential advantages over the existing methods for peptide quantification, and represent a basis from which more general methodologies might be developed.

## Materials and methods

### Materials

HPLC-grade acetonitrile, HPLC-grade water and tris(hydroxymethyl)aminomethane (Tris) were purchased from Fisher Scientific (Loughborough, UK); Ac-Tyr-OEt monohydrate (purity, HPLC 99.3 %, water content, Karl Fischer titration 5.94 %) was purchased from Santa Cruz Biotechnology (Heidelberg, Germany); dimethylsulfoxide (DMSO), 5,5′-dithiobis(2-nitrobenzoic acid) (DTNB), formic acid, reduced l-glutathione (GSH; purity, HPLC 99.3 %, purity, iodine titration 99 %) and iodoacetamide were purchased from Sigma-Aldrich (Dorset, UK); hydrochloric acid was purchased from VWR International (Leicestershire, UK); polystyrene 96-well plates were purchased from Greiner Bio One (Gloucester, UK); iT3 (ALVLIAFAQYLQQCPFEDHVK, in which valine is fully labelled with ^13^C and ^15^N; HPLC-UV purity > 95 %) was purchased from a commercial source (details available from the authors on request).

### Reversed-phase HPLC

Reversed-phase HPLC was carried out using an Agilent 1100 system consisting of a binary pump, an online de-gasser, a manual injection valve, a heated column compartment, a diode array detector (DAD) and a fluorescence detector (FD). Eluent A was 0.1 % (*v*/*v*) aqueous formic acid and eluent B was 0.1 % (*v*/*v*) formic acid in acetonitrile. For quantitative analyses, injections were performed by overfilling a 50- or 20-μL sample loop with at least three volumes of sample. For qualitative analyses, the loop size was 100 μL or 1 mL and a partial-fill method was used. In all cases, the stationary phase was C5-functionalised silica with a particle size of 5 μm (Supleco BIO Wide Pore, Sigma-Aldrich Co. Ltd, Dorset, UK). Various different packed columns were used, either individually or in pairwise combinations depending on the requirements of the analysis (see Table 1). The column temperature was 25 °C, the flow rate was 1.5 mL^−1^ and, unless otherwise stated, all substances were eluted isocratically at 30 % of eluent B. The DAD was used to monitor UV absorbance of the column effluent at two fixed wavelengths (210 and 278 nm). For HPLC-FD_Tyr_, detection of Tyr fluorescence was achieved using an excitation wavelength (*λ*_ex_) of 278 nm, an emission wavelength (*λ*_em_) of 312 nm and a PMT gain of 10. Chromatograms were analysed in Agilent ChemStation using auto-integration where possible. In cases where chromatographic irregularities prevented the use of auto-integration, a peak area was taken as the mean from three manual integrations. Absorption and fluorescence spectra were acquired using the DAD and the FD, respectively. For fluorescence emission scanning, *λ*_ex_ was 278 nm; for fluorescence excitation scanning, *λ*_em_ was 312 nm. For each substance, spectra recorded over a range of relevant retention times (the full peak width at half-maximum height) were averaged in ChemStation. Fraction collection was performed manually on the basis of real-time absorbance measurements at the DAD. The time offset between the DAD and the fraction collection was determined using a tracer substance (2-nitro-5-thiobenzoic acid).

**Table 1:** Column assemblies used for reversed-phase HPLC of peptides and Ac-Tyr-OEt

Column assembly	Column 1^a^			Column 2^a^		
	Product no.	Length (mm)	I.D. (mm)	Product no.	Length (mm)	I.D. (mm)
A	568472-U	20	4.0	568421-U	100	4.6
B	568472-U	20	4.0	–	–	–
C	568472-U	20	4.0	568420-U	50	4.6
D	568421-U	100	4.6	–	–	–
E	568423-U	250	4.6	–	–	–
F	568472-U	20	4.0	568472-U	20	4.0

Product numbers correspond to entries in the Sigma-Aldrich catalogue

*I.D.* inner diameter

^a^For pairwise combinations, numbering corresponds to the order of serially connected columns in the direction of flow

### Mass spectrometry

Electrospray ionisation mass spectrometry (ESI-MS) was used for the characterisation of Cam-iT3 and for the identification of peptidic impurities in the commercial iT3 preparation. HPLC fractions containing the peptide(s) of interest were infused at 10 μL min^−1^ (syringe pump) into the ESI source of a Thermo LTQ-XL linear ion trap mass spectrometer (Thermo Fisher Scientific, Hemel Hempstead, UK). Samples were ionised in positive mode using a capillary temperature of 300 °C, a capillary voltage of 19 V, a spray voltage of 4 kV and a sheath gas (nitrogen) flow rate of 8 arb. units. The ion trap was operated under 2.3 × 10^−5^ Torr of helium, which functioned as both the damping gas and the collision gas (see below). For tandem mass spectrometry (MS/MS), the isolation width was 1 *m*/*z*, and collision-induced dissociation was achieved using a normalised collision energy of 20 %. Spectra were acquired and evaluated using the Xcalibur software (Thermo Fisher Scientific).

### Preparation of Cam-iT3

Cam-iT3 was prepared essentially as described by Li et al. [13]. Full details and characterisation data can be found in the Electronic Supplementary Material (ESM).

### Preparation of Ac-Tyr-OEt standard solutions

Ac-Tyr-OEt monohydrate was dissolved at 10 mM in a 30 % (*v*/*v*) aqueous solution of acetonitrile containing 0.1 % (*v*/*v*) formic acid. Using the same acidified acetonitrile solution as a diluent, two sets of replica standards were prepared independently from the 10-mM stock.

### Estimation of lower limits of linear dynamic ranges

Eight concentrations of Ac-Tyr-OEt were analysed using HPLC-UV_278_-FD_Tyr_ (i.e. HPLC with simultaneous detection of UV_278_ and Tyr fluorescence; see ESM Figures S3 and S4). UV_278_ and FD_Tyr_ peak areas for the four most concentrated standards (5, 10, 20 and 50 μM) were plotted as functions of concentration, and in each case, linearity was confirmed using ordinary least squares (OLS) linear regression analysis (see ESM). The equations of the regression lines were used to predict peak areas for the lower concentration range (1 μM, 500 nM, 100 nM and 10 nM), and the deviations of the observed values from the expected values were used to calculate percentage errors. The lower limit of linearity was taken as the concentration below which the error became greater than 5 %.

### Determination of relative fluorescence responses

The relative fluorescence response of a substance (*R*_substance_) is defined according to Eq. 1, in which the peak area terms refer to HPLC-UV_278_-FD_Tyr_ data from one or more injections. In practice, two slightly different methods were used: for Ac-Tyr-OEt, the FD_Tyr_ peak areas for four concentrations were regressed on their corresponding UV_278_ peak areas (OLS linear regression), and *R*_Ac-Tyr-OEt_ was taken as the slope of the regression line; for the peptides, only the highest available concentrations were analysed, and *R*_peptide_ (i.e. *R*_Cam-iT3_ or *R*_iT3_) was taken as the mean from three replicates.

1 $$ {R}_{\mathrm{substance}}=\frac{{\mathrm{FD}}_{\mathrm{Tyr}}\ \mathrm{peak}\;\mathrm{area}}{{\mathrm{UV}}_{278}\ \mathrm{peak}\;\mathrm{area}} $$

### Quantification of Cam-iT3 relative to Ac-Tyr-OEt

In order to obtain comparable UV_278_ and FD_Tyr_ responses for Ac-Tyr-OEt and Cam-iT3, conditions in the detectors’ flow cells had to be kept constant across all measurements (hence isocratic elution of all substances with the same mobile phase). Under these conditions, however, no one column assembly performed well for both substances. Therefore, an approach was taken whereby an optimal assembly was configured for each substance, and the assemblies were manually switched or reconfigured according to the substance under analysis. Thus, for quantification of Cam-iT3, the first step was to analyse a set of Ac-Tyr-OEt standards (5, 10, 20 and 50 μM) using column assembly A (see Table 1). Next, the peptide solution of unknown concentration was analysed in triplicate using column assembly B. Finally, to account for any instrumental drift, the second set of Ac-Tyr-OEt standards was analysed, again using column assembly A. OLS linear regression and associated statistical tests were used to determine the functional relationship between Ac-Tyr-OEt concentration and FD_Tyr_ response (see ESM). Further analytical procedures are described in ‘Results and Discussion’.

### Testing for adsorptive losses of Ac-Tyr-OEt and Cam-iT3 in HPLC-FD_Tyr_

Each substance was chromatographed on a pair of relevant column assemblies (assemblies D and E for Ac-Tyr-OEt, assemblies B and F for Cam-iT3; see Table 1). All concentrations were within the method’s linear dynamic range. For each substance/assembly, the FD_Tyr_ responses from multiple injections (*n* ≥ 3) were measured. The analyses were blocked according to substance, but randomised with respect to column assembly. If a lower mean response was observed for a longer column (i.e. implying loss), the significance of the result was tested using a one-tailed independent samples *t* test in Microsoft Excel 2010. On the basis of additional results obtained in our laboratory (data not shown), other potential sources of loss (e.g. adsorption to vials and tubing) were assumed to be insignificant.

### Proof of principle using independent assays

iT3 was quantified relative to GSH using a microplate assay based on the method of Ellman [14]. This assay was developed so that GSH and iT3 could be analysed under mutually compatible conditions. Stock solutions of iT3 were prepared in DMSO, and GSH standard solutions were prepared in a degassed 1:1 mixture of Tris-HCl buffer (0.1 M, pH 8.0) and acetonitrile. Two replica sets of standards (12.5, 25, 50, 75 and 100 μM) were prepared, with each set derived from an independent weighing of solid GSH. In a 96-well microplate, the GSH standards and the iT3 stocks were combined with appropriate diluents (DMSO and/or Tris-buffered acetonitrile solution) so that all solutions had the same final volume (100 μL) and the same composition (nine parts buffer, nine parts acetonitrile and two parts DMSO). Thiol groups were derivatised by adding 100 μL of 0.5 mg mL^−1^ DTNB, equilibrated at pH 8.0 in Tris-buffered acetonitrile solution, to each well. The microplate was covered, incubated at ambient temperature for 5 min and then analysed spectrophotometrically at 405 nm using a Biotek ELx800 plate reader (BioTek Instruments, Inc., Bedfordshire, UK). In the absence of DTNB, neither iT3 nor GSH had any significant absorbance at 405 nm, indicating that the raw absorbance data did not need to be corrected. The functional relationship between GSH concentration and absorbance was established using OLS linear regression (see ESM), and the equation of the regression line was used to predict the concentrations of three iT3 stocks (different concentrations but same order of magnitude) that had each been analysed in triplicate (coefficient of variation < 3 %). For the corresponding HPLC-FD_Tyr_ measurements, the stocks were diluted 100 times into 30 % (*v*/*v*) aqueous acetonitrile containing 0.1 % (*v*/*v*) formic acid and analysed as described for Cam-iT3.

### Characterisation of peptidic impurities

Approximately 10 μg of the iT3 preparation was analysed via HPLC-FD_Tyr_ with offline ESI-MS. For this particular purpose, column assembly C provided optimal separation and peak definition. Fractions of HPLC effluent containing fluorescent material were infused directly into the mass spectrometer as described above. Putative peptide peaks were resolved in selected ion monitoring mode, and charge states were assigned on the basis of peak separation in the isotopic ion cluster. Based on the putative monoisotopic signals, initial assignments were made by identifying matches from a list of values calculated for plausible impurities (see ESM). Assignments for which the calculated *m*/*z* was within ±0.2 *m*/*z* of the observed value were submitted for validation using MS/MS data. The *m*/*z* values of all fragment ions with relative abundance >2 % were checked against a list of calculated values comprising theoretical peptide backbone fragments (singly, doubly and triply protonated *a*-, *b*-, *y*- and *z*-type product ions [15]). Assignments for which the calculated *m*/*z* was within ±0.5 *m*/*z* of the observed value were used to verify parts of the sequence, and the candidate sequence best represented by the data was selected as the likely identity of the substance.

## Results and discussion

The choice of Ac-Tyr-OEt as the calibrant was motivated by a report from Edelhoch [12], who observed very similar *ε*_275.5_ values for Ac-Tyr-OEt and Gly-Tyr-Gly (1500 and 1470 M^−1^ cm^−1^, respectively). This implied that a known relationship between Ac-Tyr-OEt concentration and UV response could be used to predict peptide concentrations (and vice versa). The error on this type of estimate would be equivalent to the difference in *ε* (approximately 2 %), and could be reduced by making the appropriate correction. For the present study, these principles seemed equally applicable to Cam-iT3, which also contains a single Tyr residue. It was considered that if the substances’ quantum yields of fluorescence were also equivalent, Ac-Tyr-OEt would recapitulate the fluorescence of Cam-iT3 as well. This reasoning led to an initial hypothesis: that a known relationship between Ac-Tyr-OEt concentration and FD_Tyr_ response could be used to predict peptide concentrations. Typically, such a relationship is established using OLS linear regression analysis, and predictions are made using the equation of the regression line. Equation 2 therefore summarises the initial hypothesis using relevant variables and regression coefficients: *r*_peptide_ is the FD_Tyr_ response of the peptide analyte, *m*_Ac-Tyr-OEt_ is the slope of a regression line for response on concentration, *b*_Ac-Tyr-OEt_ is the y-intercept and *c*_peptide_ is the predicted concentration.

2 $$ {c}_{\mathrm{peptide}}=\frac{r_{\mathrm{peptide}} - {b}_{\mathrm{Ac}\hbox{-} \mathrm{T}\mathrm{y}\mathrm{r}\hbox{-} \mathrm{O}\mathrm{E}\mathrm{t}}}{m_{\mathrm{Ac}\hbox{-} \mathrm{T}\mathrm{y}\mathrm{r}\hbox{-} \mathrm{O}\mathrm{E}\mathrm{t}}} $$

**Fig. 1:**
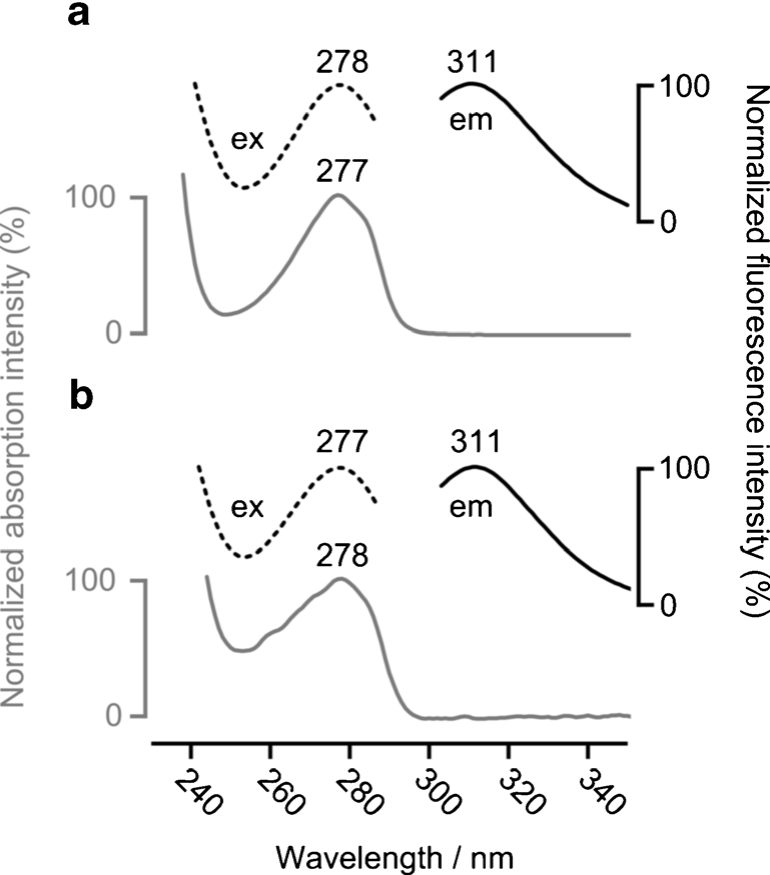
Absorption and fluorescence spectra of **a** Ac-Tyr-OEt and **b** Cam-iT3. Normalised intensity is relative to the peak of interest. For the fluorescence excitation spectrum (*dashed line*), *λ*_em_ was 312 nm; for the emission spectrum (*solid line*), *λ*_ex_ was 278 nm

As a first test, the two substances were analysed in a series of UV/visible and fluorescence scanning experiments. Each substance was chromatographed using isocratic elution with the same mobile phase (30 % of eluent B), and spectra were recorded using the DAD and the FD. Cam-iT3 and Ac-Tyr-OEt displayed near-identical maxima in their absorbance, fluorescence excitation and fluorescence emission spectra (Fig. 1). The absorption maxima were consistent with relevant examples from the literature [11, 16]. The fluorescence emission maxima were 9 nm higher than a literature example (Leu-Tyr-Leu), probably as a result of differences in the conditions under which the measurements were made.

Next, the fluorescence responses of Cam-iT3 and Ac-Tyr-OEt were compared. Assuming that *ε*_278_ (i.e. the value of *ε* at the measured wavelength of maximum absorbance) would be constant across all substances, relative responses (*R*_substance_) were calculated from UV_278_ and FD_Tyr_ peak area data as described in ‘Materials and Methods’. Using *R*-values, it was then possible to test the validity of the initial hypothesis. For the hypothesis to be supported, a ratio of these values, *R*_Ac-Tyr-OEt_/*R*_Cam-iT3_, should equal unity: if *R*_Ac-Tyr-OEt_/*R*_Cam-iT3_ < 1, Eq. 2 would overestimate *c*_peptide_; conversely, if *R*_Ac-Tyr-OEt_/*R*_Cam-iT3_ > 1, *c*_peptide_ would be underestimated. The ratio determined for Cam-iT3 was 0.342, meaning that the initial hypothesis was not supported because Cam-iT3 was more fluorescent than Ac-Tyr-OEt. In the absence of literature data from directly comparable systems, we do not attempt to rationalise this result here, but we acknowledge that it is sufficiently interesting to warrant further investigation.

It was now considered that the prediction accuracy could be improved by applying *R*_Ac-Tyr-OEt_/*R*_peptide_ as a correction factor. This formed the basis of a modified hypothesis based on the use of Eq. 3 to obtain *c*_peptide_.

3 $$ {c}_{\mathrm{peptide}}=\frac{r_{\mathrm{peptide}}-{b}_{\mathrm{Ac}\hbox{-} \mathrm{T}\mathrm{y}\mathrm{r}\hbox{-} \mathrm{O}\mathrm{E}\mathrm{t}}}{m_{\mathrm{Ac}\hbox{-} \mathrm{T}\mathrm{y}\mathrm{r}\hbox{-} \mathrm{O}\mathrm{E}\mathrm{t}}}\times \frac{R_{\mathrm{Ac}\hbox{-} \mathrm{T}\mathrm{y}\mathrm{r}\hbox{-} \mathrm{O}\mathrm{E}\mathrm{t}}}{R_{\mathrm{Cam}\hbox{-} \mathrm{iT}3}} $$

In implementing the above concepts, Ac-Tyr-OEt standard concentrations (5 to 50 μM) were selected based on the expected concentration of purified Cam-iT3 in the HPLC effluent (20 μM). The fluorescence response was linear over the concentration range of interest (see ESM), but became non-linear below 5 μM. From this result alone, HPLC-FD_Tyr_ did not appear to offer any advantage over a simpler HPLC-UV_278_ method, but clear benefits were observed when small amounts of peptide were analysed: in addition to higher sensitivity (see ESM Fig. S9), HPLC-FD_Tyr_ also provided more reproducible peak areas when the peptide concentration was near the lower limit of the linear dynamic range (e.g. coefficient of variation for triplicate analysis, 0.9 % for HPLC-FD_Tyr_ versus 6.4 % for HPLC-UV_278_).

Next, the validity of the ‘generic calibrant’ approach was considered from a practical point of view. Given that the method uses different column assemblies and fluorescent substances interchangeably, analyte-sorbent interactions represented a potential source of error. Specifically, it was hypothesised that an analyte could be lost to the stationary phase by adsorption, resulting in an inverse relationship between FD_Tyr_ response and column length. To test this, the effect of column assembly on FD_Tyr_ response was measured for each substance. For Ac-Tyr-OEt, increasing the column length by 150 % (assembly D → assembly E) did not cause any reduction in the FD_Tyr_ response. For Cam-iT3, doubling the length (assembly B → assembly F) caused the chromatographic peak to broaden considerably, making it difficult to assign a baseline. The mean response was slightly dampened (−3.8 %), but this was not statistically significant (one-tailed independent samples *t* test, *P* > 0.1). Hence, it appeared that analyte-sorbent interactions should not be a source of error.

**Fig. 2:**
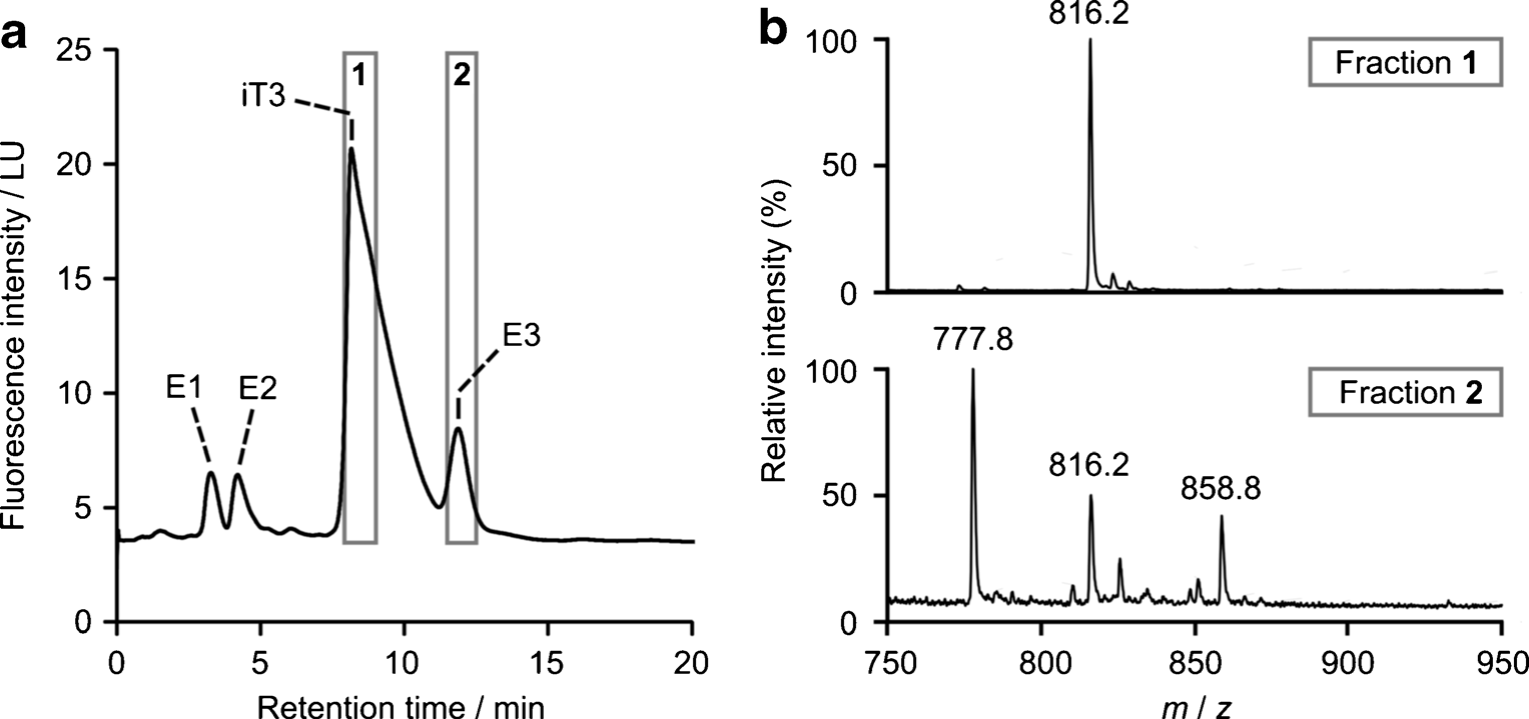
Analysis of fluorescent components in a commercial preparation of iT3: **a** chromatogram from HPLC-FD_Tyr_. *iT3* accounts for the majority of the observed fluorescence, but extraneous peaks (*E1*–*E3*) were also present. In this experiment, two fractions (*1* and *2*) were collected. **b** ESI mass spectra from infusion of the two fractions. Components *E1* and *E2* were analysed in a separate experiment (see ESM)

As an overall test of the modified hypothesis, a set of concentrations predicted using Eq. 3 were compared to an independent set of predictions for the same samples but obtained via a different method (Ellman assay [14]). Since the Ellman assay quantifies thiol groups, the unmodified commercial iT3 peptide was used instead of Cam-iT3. The fluorescence response of iT3 (*R*_Ac-Tyr-OEt_/*R*_iT3_ = 0.348) was similar to that of Cam-iT3, and apart from interacting more strongly with the stationary phase, its chromatographic behaviour was also similar. The concentrations of three iT3 solutions predicted from Tyr fluorescence (199, 393 and 612 μM) agreed well with corresponding predictions from the Ellman assay (199, 408 and 613 μM, respectively). In the FD_Tyr_ chromatograms of iT3, however, we noticed three extraneous peaks (Fig. 2a, peaks E1, E2 and E3), which we investigated further. It was considered that these peaks might be due to peptidic impurities related to iT3 (e.g. deletion sequences [5, 6]), which could potentially interfere with validation. For example, if an amino acid residue other than Cys or Tyr were deleted, one might observe a false positive response in the Ellman assay and an extraneous peak in HPLC-FD_Tyr_. The FD_Tyr_ response could be corrected by including the area under the extraneous peak, but this would require the impurity’s *R*-value to be known.

After using offline ESI-MS to confirm that the major chromatographic component was iT3 (Fig. 2b, ESM Fig. S12a and Table S6), attention was focused on the major extraneous peak (E3). Noting that, like iT3, this component was accompanied by a putative peptide bond UV response (ESM Fig. S10), we conducted further analyses using offline ESI-MS and MS/MS. Two [M + 3H]^3+^ ions corresponding to peptidic impurities were identified in the eluate (Fig. 2b, ESM Fig. S12 and Table S6): one was lighter than iT3 (*m*/*z* = 777.3) and the other was heavier (*m*/*z* = 858.3). iT3 itself (*m*/*z* = 815.6) was also observed in the same fraction, probably from the tail of the major chromatographic peak. The light impurity was 115 Da lighter than iT3, suggesting deletion [5, 6] of aspartic acid. This finding was supported by MS/MS analysis (ESM Fig. S13a), which localised the missing 115 Da to within an eight-residue sequence at the *C*-terminus of iT3 (CPFEDHVK). The heavy impurity was 128 Da heavier than iT3, suggesting insertion [5, 6] of an additional amino acid residue (glutamine or lysine). The MS/MS data were consistent with an iT3 analogue in which the internal sequence LQQC had been expanded to LQQQC (see ESM Fig. S13b). Despite observing no evidence of peptide bonds for E1 or E2 (ESM Fig. S10), these minor components were also checked using offline ESI-MS (ESM Fig. S11). No peptidic substances were detected under E1, but under E2, we found three more putative peptides. One of these (*m*/*z* 792.4; ESM Figs. S11 and S12b) was unambiguously identified by MS/MS as a deletion sequence lacking the *N*-terminal alanine residue (ESM Fig. S14). The important thing to note about the peptidic impurities is that they all contain cysteine. Thus, a proportion of the extraneous FD_Tyr_ responses could be linked to false positive responses in the Ellman assay. Since the impurities’ UV_278_ responses were all too low for specific *R*-values to be calculated, the only way of correcting for them was to assume uniform *R* across all substances (i.e. *R*_impurity_ = *R*_iT3_). When the appropriate corrections were applied, the HPLC-FD_Tyr_ results appeared to overestimate the concentration of relevant peptides by approximately 22 %. Considering the assumption regarding *R*_impurity_, however, the magnitude of this error should be interpreted with caution.

## Conclusions

A fluorescence-based method for quantifying Cam-iT3, a Tyr-containing peptide derived from the sequence of human serum albumin, has been developed and tested. Using the method, it was demonstrated that the concentration of Cam-iT3 (or iT3, a related peptide) could be readily estimated, even in the presence of peptidic impurities. Unfortunately, however, these impurities interfered with attempts to fully validate the method. The available data suggest that the ‘generic calibrant’ approach might fractionally overestimate the true peptide concentration, and further development may be required to improve its accuracy. Despite this apparent limitation, the approach still offers advantages over many of the methods used routinely for peptide quantification. Moreover, it is anticipated that a general method for the quantification of peptides with intrinsic fluorescence could emerge from this work.

## Electronic supplementary material

ESM 1 (PDF 2334 kb)
