# Supplementary material for: Quantification of a peptide standard using the intrinsic fluorescence of tyrosine
Source: Anal Bioanal Chem. 2016 Feb 15;408:2187–93. doi: 10.1007/s00216-016-9334-1 (PMC4799795; doi:10.1007/s00216-016-9334-1)
Supplement: Supplementary file 1 — (PDF 2334 kb) [file 216_2016_9334_MOESM1_ESM.pdf]

Analytical and Bioanalytical Chemistry

Electronic Supplementary Material

Quantification of a peptide standard using the intrinsic fluorescence of tyrosine

George W. Preston, David H. Phillips

Preparation and Characterisation of Cam-iT3

Cam-iT3 was prepared essentially as described by Li *et al.*[1] In a 0.5-mL microcentrifuge tube, iodoacetamide (0.38 mg, 2.0 µmol) in water (50 µL) was combined with iT3 (0.10 mg, 41 nmol) in DMSO (50 µL). Following vortex mixing, the reaction was incubated at 25 °C on a heated shaker unit for 24 h in the dark. The crude product was diluted two-fold into 30% (v/v) aqueous acetonitrile containing 0.1% (v/v) formic acid, and purified by reversed-phase HPLC (column assembly 'A'; see Materials and Methods). 100-µL loop-fill injections of the mixture were chromatographed using gradient elution (see Table S1) at a flow rate of 1.0 mL min^{-1}. Eluates containing the putative product were pooled, and purity was evaluated by re-chromatographing 100 µl of this pooled material (Fig. S1). The major component (> 97% of UV_{210} peak area) eluted earlier than unmodified iT3. For ESI-MS (see Fig. S2), the purified material was diluted to 4% (v/v) with a solution of 0.1% (v/v) formic acid in 40% (v/v) aqueous acetonitrile. Cam-iT3 was identified from an isotopic cluster of $[M + 2H]^{2+}$ ions (Fig. S2, part b): *m*/*z* calc'd for $[^{12}C_{106}{}^{13}C_{10}H_{178}{}^{14}N_{26}{}^{15}N_{2}O_{31}S]^{2+}$: 1251.66; found: 1251.68.

Table S1. Timetable for gradient elution of Cam-iT3 and related impurities.

Time / min	Fraction of eluent B (%)
0.0	30
0.5	30
8.5	38
9.0	95
10.0	95
10.5	30
14.0	30

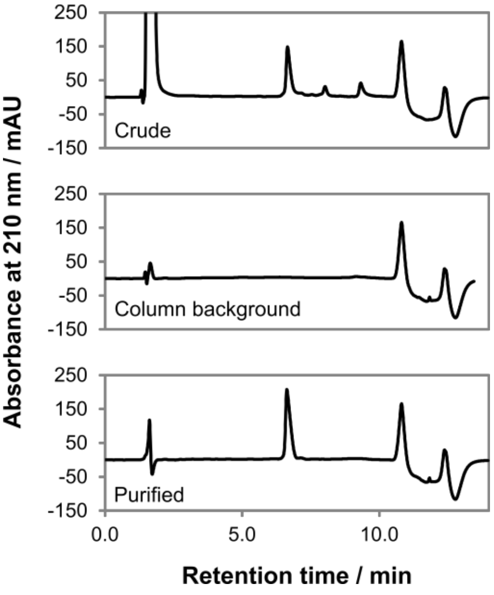

Figure S1. Successful purification of Cam-iT3 as evidenced by analytical HPLC-UV. The purified material (bottom panel) was completely free of the major impurities present in the crude product (top panel), and all other observed peaks were accounted for by the column background (middle panel).

(a)

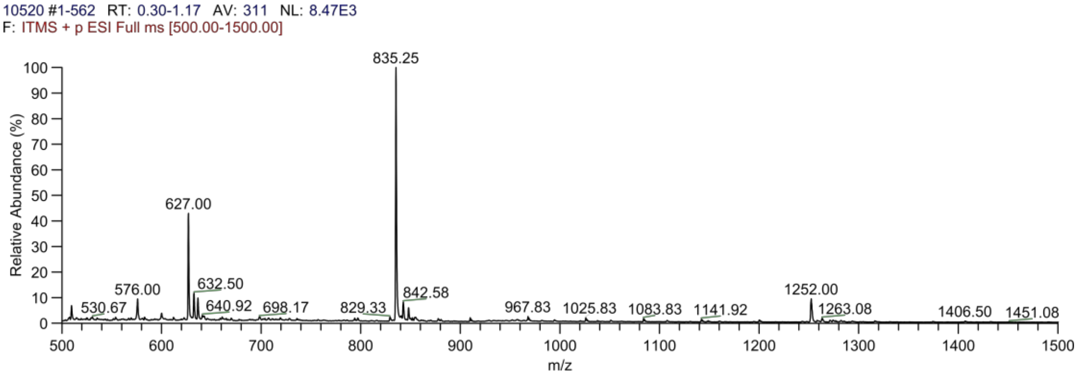

(b)

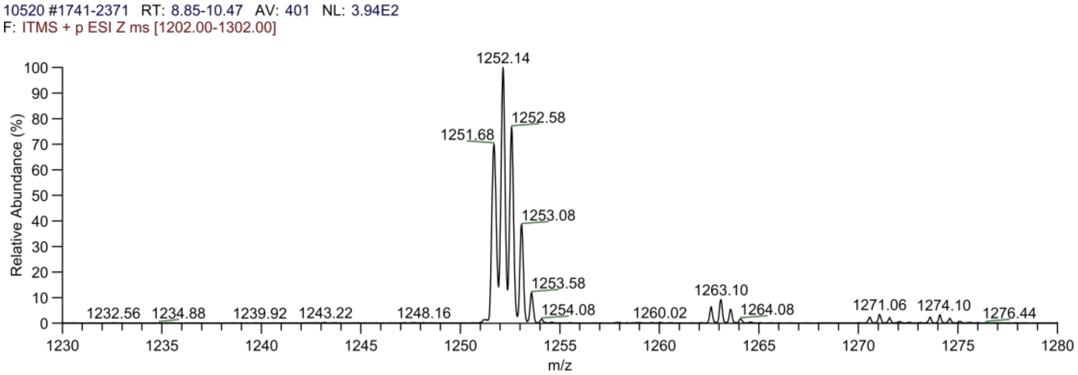

Figure S2. ESI mass spectrometry of purified Cam-iT3: (a) Spectrum showing the charge-state distribution of Cam-iT3. The signals at m/z = 627, m/z = 835 and m/z = 1252 were assigned as $[M + 4H]^{4+}$, $[M + 3H]^{3+}$ and $[M + 2H]^{2+}$, respectively; (b) resolved isotopic cluster of $[M + 2H]^{2+}$ ions from which the identity of Cam-iT3 was confirmed.

Calibration Data and Linear Regression Analysis

*HPLC-**<u>UV₂₇₈</u>**-FD_{Tyr}*

Concentrations used to investigate the linear dynamic range spanned four orders of magnitude. Hence, $\log_{10}$ transformation of the variables was appropriate for visualising the entire range (Fig. S3).

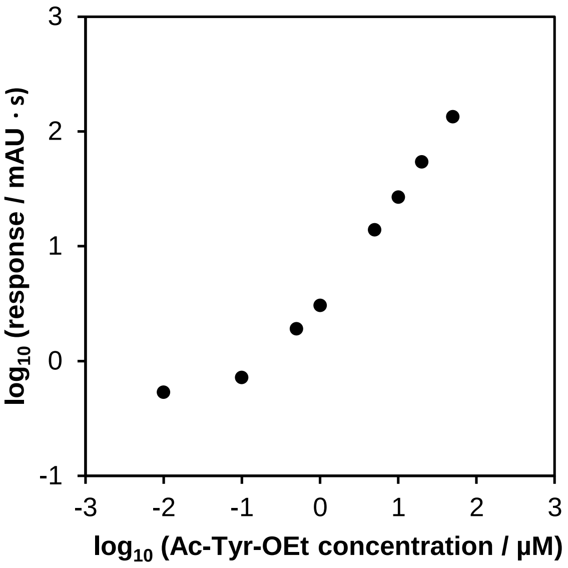

Figure S3. Scatter plot of $\log_{10}$-transformed calibration data for visualisation of the HPLC-UV_{278} dynamic range

*HPLC-UV_{278}-**FD$_{Tyr}$***

As for UV_{278} detection (see above), a qualitative assessment of the linear dynamic range was carried out using $\log_{10}$-transformed data (Fig. S4). Then, using untransformed data, the lower limit of the linear dynamic range was determined quantitatively as described in Materials and Methods. This analysis indicated that only data for the four highest concentrations of Ac-Tyr-OEt (5 µM, 10 µM, 20 µM and 50 µM) should be used for calibration. The eight untransformed[*] data (four concentrations × two replicates) were used for linear regression analysis and associated statistical tests (see Fig. S5, Fig. S6, Table S2 and Table S3). Linear regression (ordinary least squares) was carried out using the Analysis TookPak in Microsoft Excel 2010. The lack-of-fit *F*-statistic[2] was calculated manually using the same software, and its significance was evaluated using a tabular *F*-distribution. This test indicated that there was no significant lack of fit.

[*] The uneven spacing of Ac-Tyr-OEt concentrations, which was an inevitable consequence of covering four orders of magnitude, meant that the data points had different degrees of influence. To mitigate potential errors in prediction accuracy, a parallel log-log regression analysis was conducted (both variables $\log_{10}$-transformed as in Fig. S4). This had the effect of spacing the data more evenly in the X-dimension, but it did not significantly affect predicted values of X; thus, the interchangeability of these results implies that different degrees of influence had no real effect on the regression line. It is simply for the sake of clarity that untransformed data are used for derivation of statistics, and for subsequent analysis of the peptide data.

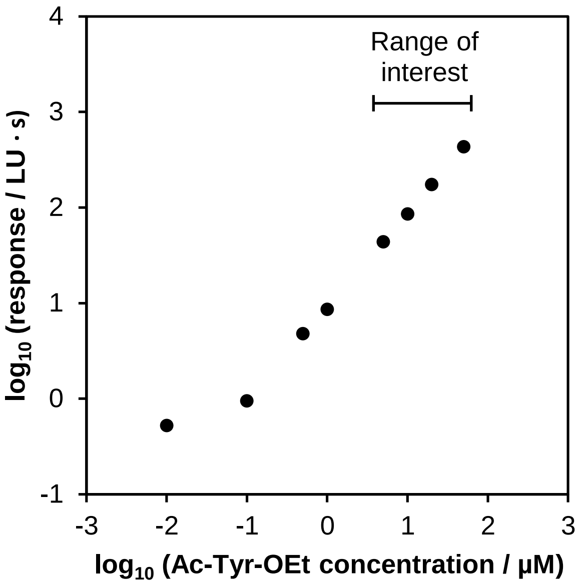

Figure S4. Scatter plot of log_{10}-transformed calibration data for visualisation of the FD_{Tyr} dynamic range. Each point represents the mean of two replicates. Only data for the four concentrations in the range of interest were used for linear regression analysis

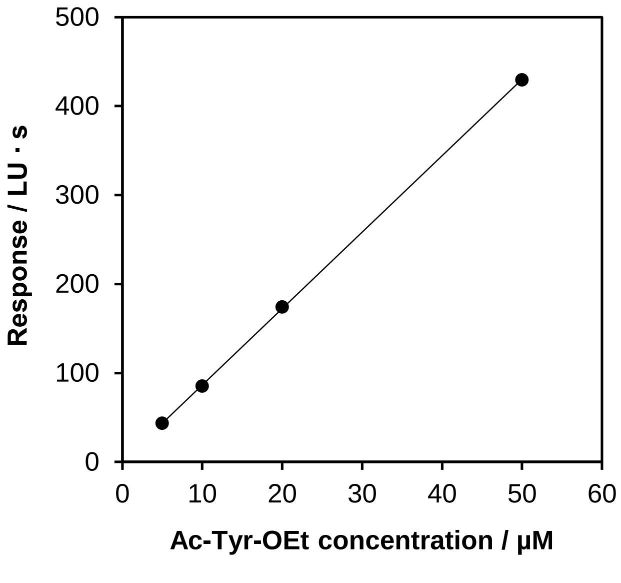

Figure S5. Linear regression analysis of four-point calibration for FD_{Tyr}: scatter plot with regression line. Untransformed data were used (see footnote on previous page). Regression coefficients and associated statistics can be found in Table S2

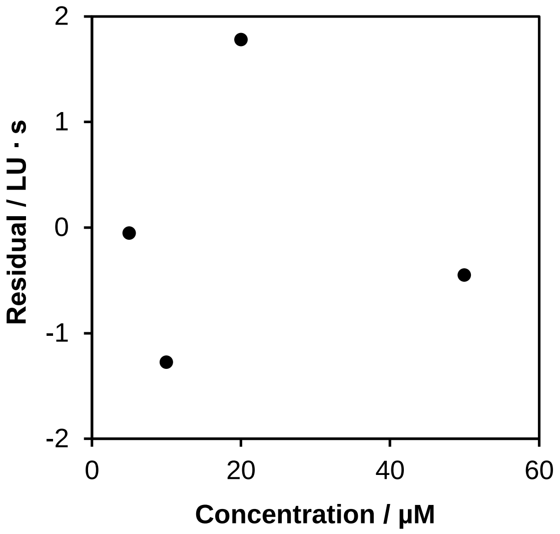

Figure S6. Linear regression analysis of four-point calibration for FD_{Tyr}: residuals plot

Table S2. Linear regression analysis of four-point calibration for FD_{Tyr}: regression coefficients and associated statistics

**Coefficient**	**Value**[a]	**Standard deviation**[a]	**Student's *t*-test**		**95% Confidence limits**[a]	
			t	***P*-value**	**Lower**	**Upper**
y-Intercept	0.681	1.25	0.547	0.639	−4.68	6.04
Slope	8.58	0.0453	189	2.79×10^{-5}	8.39	8.78

[a] Units as per Figures S5 and S6

Table S3. Linear regression analysis of four-point calibration for FD_{Tyr}: coefficient of determination, ANOVA and lack-of-fit F-test

**Statistic**	**Value**	***P*-value**
r^2	0.99994	-
ANOVA $F_{1,2}$	35898	2.79×10^{-5}
Lack-of-fit $F_{2,4}$	0.387	> 0.1

Ellman Assay

The methods used to analyse absorbance data from the Ellman assay were exactly as described for HPLC-FD_{Tyr} (see above), only in this case there were ten data (five concentrations × two replicates). The results from linear regression analysis can be found in Fig. S7, Fig. S8, Table S4 and Table S5.

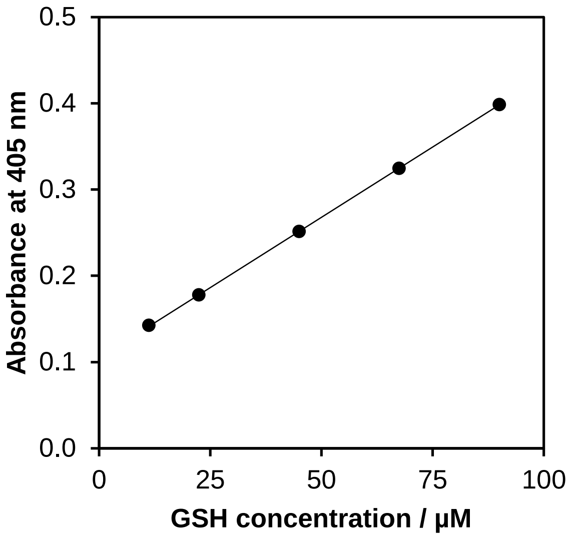

Figure S7. Linear regression analysis of five-point calibration for the Ellman assay: scatter plot with regression line

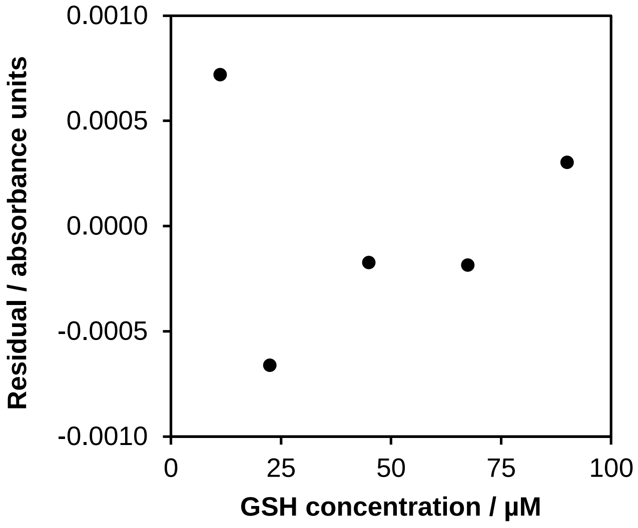

Figure S8. Linear regression analysis of five-point calibration for the Ellman assay: residuals plot

Table S4. Linear regression analysis of five-point calibration for the Ellman assay: regression coefficients and associated statistics

Coefficient	Value[a]	Standard deviation[a]	Student's *t*-test		95% Confidence limits[a]	
			t	*P*-value	Lower	Upper
y-Intercept	0.105	5.23×10^{-4}	201	2.73×10^{-7}	0.103	0.107
Slope	0.00326	9.45×10^{-6}	345	5.38×10^{-8}	0.00323	0.00329

[a] Units as per Figures S7 and S8

Table S5. Linear regression analysis of five-point calibration for the Ellman assay: coefficient of determination, ANOVA and lack-of-fit *F*-test

Statistic	Value	*P*-value
r^2	0.99998	-
ANOVA $F_{1,3}$	118815	5.38×10^{-8}
Lack-of-fit $F_{3,5}$	0.118	> 0.1

Supplementary Chromatographic Data

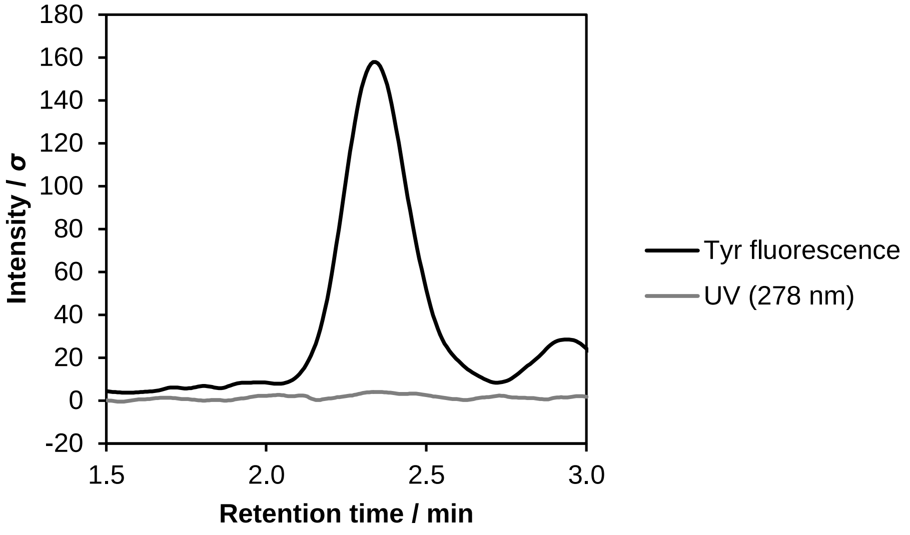

Figure S9. Comparison of UV_{278} and FD_{Tyr} responses for a concentration of iT3 that was close to the lower limit of the HPLC-FD_{Tyr} linear dynamic range. Retention times are corrected to account for the time offset between the DAD and the FD. Intensities are expressed in terms of standard deviation (σ) for the baseline noise of the respective trace

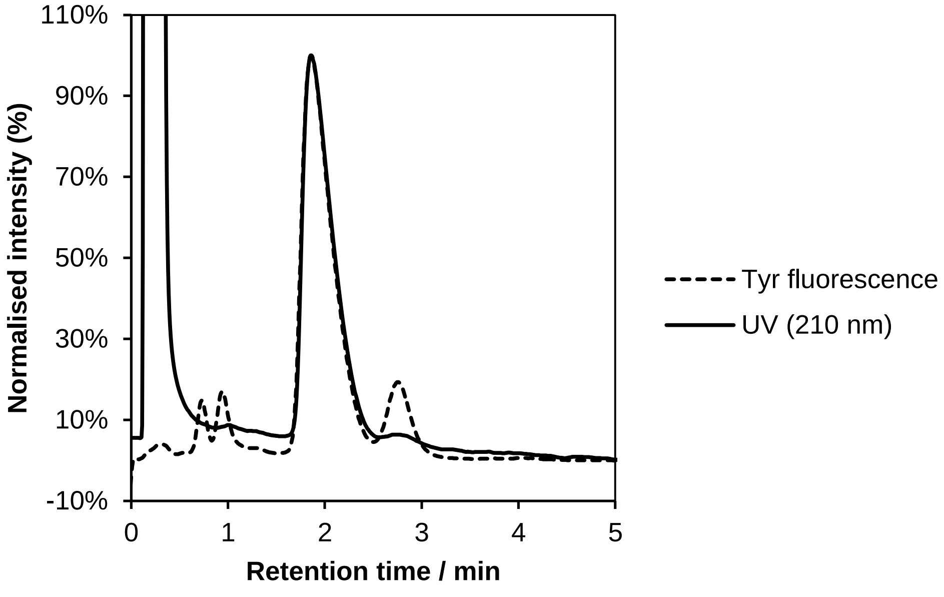

Figure S10. Comparison of UV_{210} and FD_{Tyr} responses for impurities in a commercial preparation of iT3. Normalised intensity is relative to the iT3 peak. Retention times are corrected to account for the time offset between the DAD and the FD

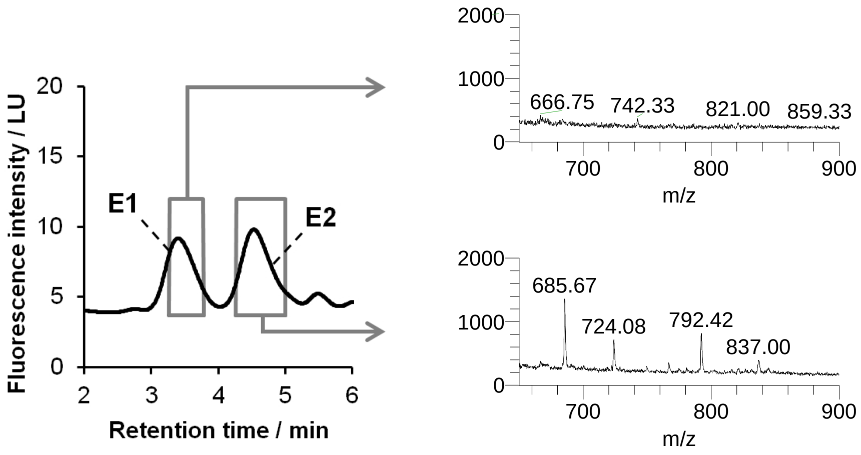

Figure S11. HPLC-FD$_{Tyr}$ and offline MS of extraneous components E1 and E2. Grey boxes on the chromatogram indicate fraction collection, and the mass spectrum obtained upon infusion of each fraction is indicated. For comparative purposes an absolute intensity scale is used.

Further Characterisation of iT3 and Related Impurities

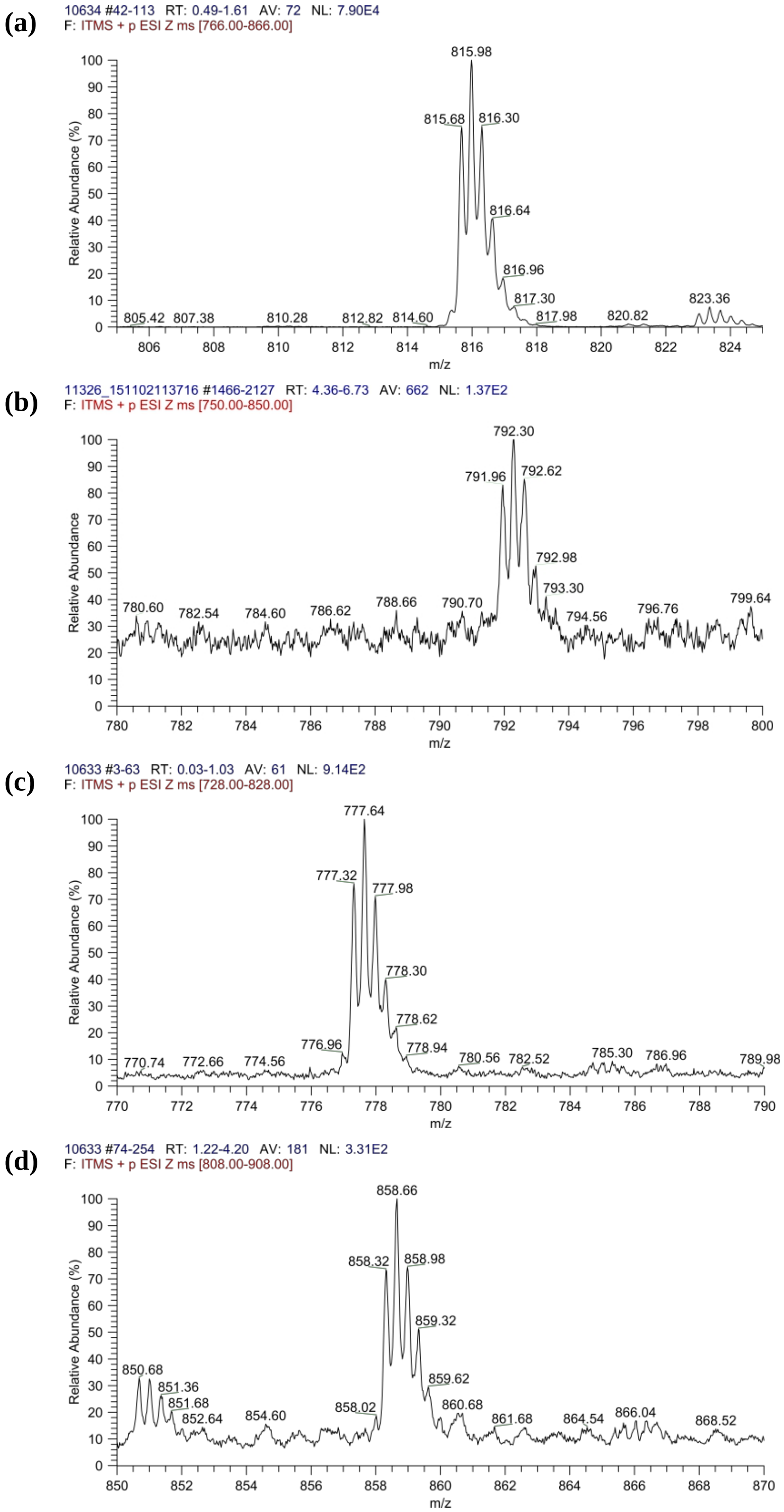

Figure S12. Resolved isotopic clusters of putative $[M + 3H]^{3+}$ ions: (a) iT3; (b) the 'light' impurity in component E2; (c) the 'light' impurity in component E3; (d) the 'heavy' impurity in component E3.

Enumeration of Plausible Peptidic Impurities

The most likely types of impurity were identified as deletion sequences (i.e., absence of ≥ 1 amino acid residue) and insertion sequences (i.e., presence of ≥ 1 additional amino acid residue).[3] The deletion or insertion of > 1 amino acid was considered sufficiently improbable to be insignificant, so the possibilities were restricted to sequences containing ± 1 amino acid. For the deletion sequences, it was considered that any of the 21 amino acids in iT3 could be absent (21 possible deletion sequences; 11 different masses). For the insertion sequences, it was considered that an additional amino acid could only be incorporated *via* unintended double coupling (i.e., two copies of the same amino acid incorporated during in a single synthetic step). Again, it was considered that this process could occur for any of the 21 amino acids in iT3 (20 different sequences, 12 different masses). Note that double coupling of Gln at either …LQQC… or …LQQC… would give the same insertion sequence (…LQQQC…), hence why there are fewer possible insertion sequences than there are amino acids in iT3.

In solid phase peptide syntheses using the 9-fluorenylmethoxycarbonyl (Fmoc) strategy, added mass can also arise from oxidation, piperidide formation or incomplete removal of protecting groups (e.g., Fmoc, *tert*-butyl or *tert*-butyloxycarbonyl).[3] The specific reagents and procedures used for the preparation of iT3 were not known, but we noted that the observed impurities were not consistent with these modifications.

Table S6. Sequence information and mass spectrometric data for iT3 and related impurities

**Substance**	**Proposed sequence**[a]	**Chemical formula of [M + 3H]$^{3+}$**	**Calculated *m/z* for [M + 3H]$^{3+}$**	**Observed *m/z***[b]
iT3	ALVLI AFAQY LQQCP FEDHV K	$[^{12}C_{104}{}^{13}C_{10}H_{176}{}^{14}N_{25}{}^{15}N_2O_{30}S]^{3+}$	815.77	815.68
E2 light impurity	LVLIA FAQYL QQCPF EDHVK	$[^{12}C_{101}{}^{13}C_{10}H_{171}{}^{14}N_{24}{}^{15}N_2O_{29}S]^{3+}$	792.09	791.96
E3 light impurity	ALVLI AFAQY LQQCP FEHVK	$[^{12}C_{100}{}^{13}C_{10}H_{171}{}^{14}N_{24}{}^{15}N_2O_{27}S]^{3+}$	777.43	777.32
E3 heavy impurity	ALVLI AFAQY LQQQC PFEDH VK	$[^{12}C_{109}{}^{13}C_{10}H_{184}{}^{14}N_{27}{}^{15}N_2O_{32}S]^{3+}$	858.45	858.32

[a] All valine residues were uniformly labelled with ^{13}C and ^{15}N; [b] see Fig. S12

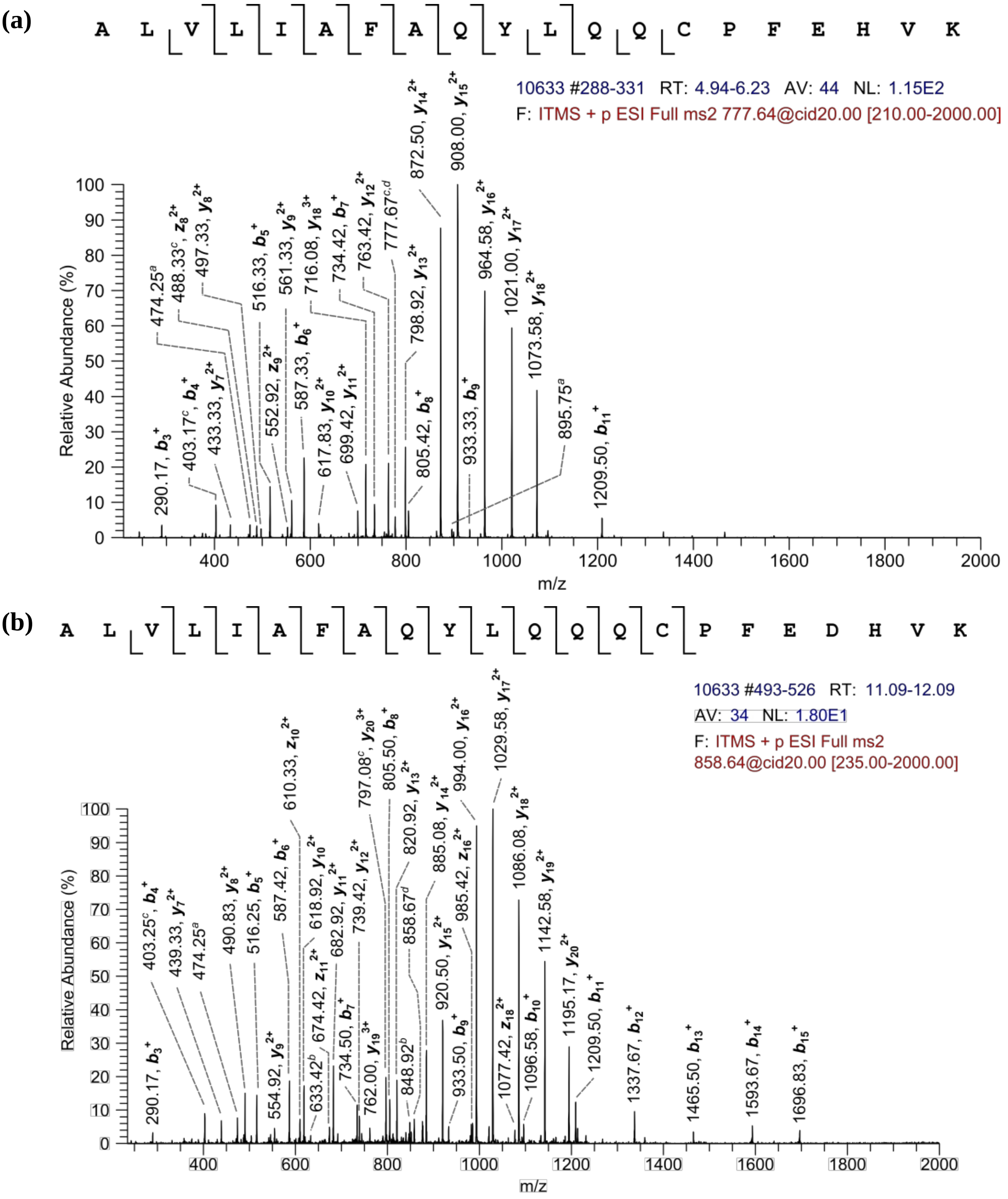

Figure S13. Tandem mass spectrometry of two impurities detected in chromatographic component E3: (a) the ‘light’ peptidic impurity; (b) the ‘heavy’ peptidic impurity. Fragments are identified using standard nomenclature.[4] The charge assigned to each fragment is also indicated. Superscript letters refer to the following additional annotations: [a] no valid assignments for this peak; [b] assignment valid but anomalous (e.g., charge inconsistent with other observed fragments of the same class); [c] multiple valid assignments, so assignment chosen on the basis of consistency with other observed fragments; [d] assigned as residual precursor ion. Above each spectrum, observed fragmentations are mapped onto the proposed sequences, with vertical lines indicating position and horizontal lines indicating charge retention

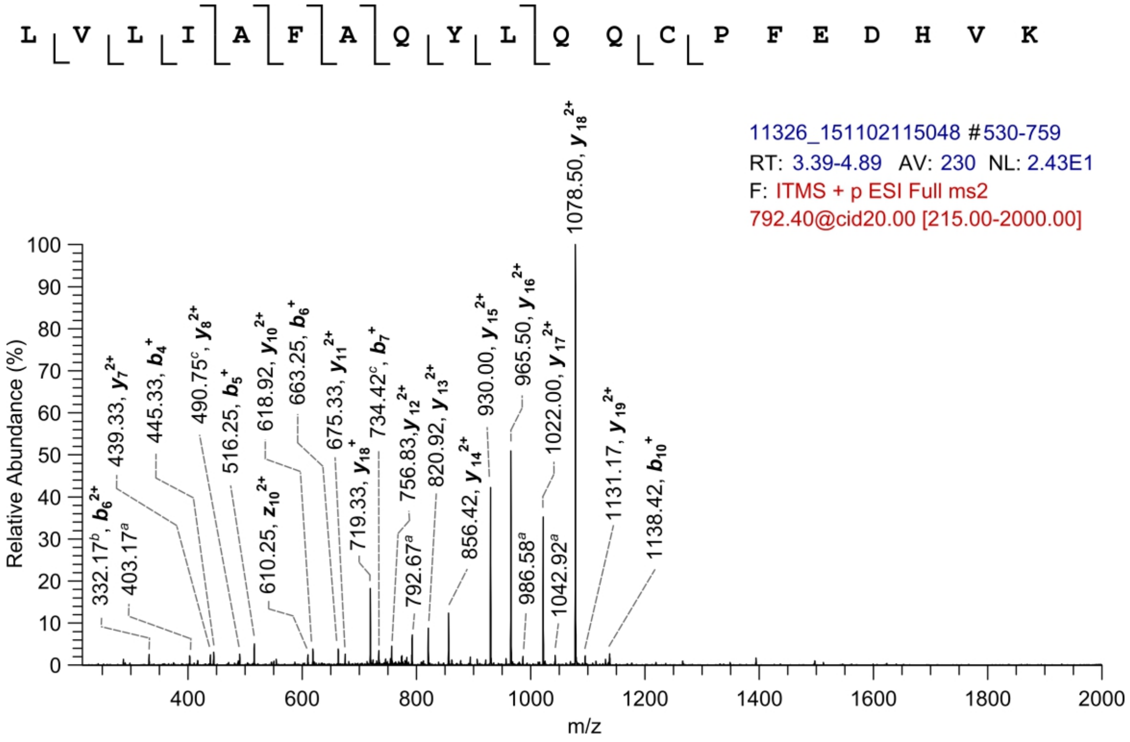

Figure S14. Tandem mass spectrometry of an impurity detected in chromatographic component E2 of the commercial iT3 preparation. The annotations and nomenclature are as per Fig. S13

References for the Supplementary Information

(1) Li, H.; Grigoryan, H.; Funk, W. E.; Lu, S. S.; Rose, S.; Williams, E. R.; Rappaport, S. M. *Mol. Cell. Proteomics* **2011**, *10*, M110.004606.

(2) Weisberg, S. *Applied Linear Regression*; John Wiley & Sons, Inc.: New York, 1980.

(3) D'Hondt, M.; Bracke, N.; Taevernier, L.; Gevaert, B.; Verbeke, F.; Wynendaele, E.; De Spiegeleer, B. *J. Pharm. Biomed. Anal.* **2014**, *101*, 2.

(4) Biemann, K. In *Methods in Enzymology*; James, A. M., Ed.; Academic Press, 1990; Vol. 193.
